# Planning of HIFU therapies of moving organs by using numerical simulation techniques

**DOI:** 10.1186/2050-5736-2-S1-A8

**Published:** 2014-12-10

**Authors:** Joachim Georgii, Caroline von Dresky, Daniel Demedts, Christian Schumann, Tobias Preusser

**Affiliations:** 1 Fraunhofer MEVIS, Bremen, Germany

## Background

We present a software prototype that allows for pre-interventional planning and simulation of focused ultrasound therapies. By using numerical simulation techniques the heating and destruction of the tissue can be predicted and thus a patient-specific treatment plan can be generated in advance. Since our simulation is amenable to respect rib shielding, organ motion as well as focal point tracing, it could in particular support the planning of liver treatments.

## Material and methods

The simulation of the ultrasound propagation is based on the hybrid angular spectrum method. The heating of the tissue is computed by solving the Pennes bioheat equation. Both simulations have been massively parallelized on graphics processing units to improve computation times. The simulation of the pressure field includes single reflection of the ultrasound beams at acoustically dense structures as the ribs. Technically, these reflections are computed by a so-called back-sweep of the spectral propagator. Moreover, motion of the tissue is incorporated in the simulation by allowing the material parameters to be updated in each simulation step. Additionally, heat transportation of moving tissue is respected in the bioheat equation solver. By adjusting the transducer parameters for electronic steering, we can simulate focal point tracing in moving organs. Based on the temperature field, we also predict the thermal damage by computing the Arrhenius damage model.

## Results

Our approach allows for the simulation of entire treatment plans composed of a set of sonications and cooling phases. For a 40s sonication simulated at a resolution of 256x256x256 pressure samples and 128x128x128 temperature samples we observe computation times of 19s if we recompute the ultrasound field 15 times per breathing cycle. Therefore, due the parallelization approach the treatment can be simulated faster than real time in moving organs.

## Conclusion

Due to the exploitation of graphics processing units, we are able to simulate HIFU treatments in moving organs faster than real time. By simulating the pressure as well as the temperature field and the thermal damage we can predict the treatment results depending on changes of the hardware parameters such as phase shifts or power per element. Based on this simulation, a detailed pre-interventional treatment planning for liver tumors becomes possible.

